# Supplementary material for: All Models are Wrong, Some are Annotated: Automating Metadata in Biomedical Repositories
Source: bioRxiv. 2026 Apr 27:2026.04.23.720371. Preprint. [Version 1] doi: 10.64898/2026.04.23.720371 (PMC13142456; doi:10.64898/2026.04.23.720371)

bioRxiv preprint doi: https://doi.org/10.64898/2026.04.23.720371; this version posted April 27, 2026. The copyright holder for this preprint (which was not certified by peer review) is the author/funder, who has granted bioRxiv a license to display the preprint in perpetuity. It is made available under aCC-BY 4.0 International license.

Supplementary File

Appendix S1. Full LLM Prompt Templates 18
Table S1. Annotation Heuristics for Manual Labeling of ModelDB Files 20
Table S2. Feature List for XGBoost. 22
Table S3. Pairwise agreement. 26
Table S4. Run-to-run agreement vs. human annotation confidence. 26
Figure S1. Confusion Matrices. 27
Figure S2. Correct Classification Overlap. 27
Figure S3. XGBoost Feature Importance. 28

Appendix S1. Full LLM Prompt Templates

GPT 5.2 / GPT mini:

You are an expert in computational neuroscience. The following is a MOD file from a NEURON computational neuroscience model. Briefly discuss the biology that the above code appears to be modeling. End by generating JSON of the form {"mechanisms": ["term1", "term2"], "type of model": "free text", "notes": "any notes of unusual things in the model"} where the terms (typically 0 or 1 but could be more represent the mechanism(s) (currents, pumps, and receptors) that are modeled in the file, chosen verbatim from the list below. When interpreting this list, HVA is high voltage activated, LT is low threshold, Rare should be interpreted as any other type of that channel that isn't covered by a more specific channel, "I Other (Rare)" is a current (e.g., Cl) that is not a Ca, K, or Na current, a prefix of R indicates a receptor (e.g., "R Other (Rare)" is a synaptic receptor that is not GABA, AMPA, or NMDA), and "Z Neither" is something that is neither a receptor nor a current (this could be a pump or it could be a utility file with many VERBATIM blocks that has no direct biological interpretation). Provide no additional explanation after the JSON."I Ca (HVA)", "I Ca (Rare)", "I Ca (T-type LT)", "I H", "I K (A-type)", "I K (Ca-activated)", "I K (Delayed Rectifier)", "I K (M-type)", "I K (Rare)", "I Na (Persistent)", "I Na (Rare)", "I Na (Slow inactivation)", "I Na (Transient)", "I Other (Rare)", "R GABA", "R Glutamate (AMPA)", "R Glutamate (NMDA)", "R Other (Rare)", "Z Neither"

GPT 5.2 + heuristics / GPT mini + heuristics

You are an expert in computational neuroscience. The following is a MOD file from a NEURON computational neuroscience model. Briefly discuss the biology that the above code appears to be modeling. You can use the following heuristics if it helps you to understand:

NET_RECEIVE: Likely receptor; discrete events rather than continuous current.

POINT PROCESS: Likely receptor but could be some other localized mechanism (e.g. neither - graded synapse, neither - gap junction).

SUFFIX: Likely ion channel but could be neither (e.g., SUFFIX NOTHING).

bioRxiv preprint doi: https://doi.org/10.64898/2026.04.23.720371; this version posted April 27, 2026. The copyright holder for this preprint (which was not certified by peer review) is the author/funder, who has granted bioRxiv a license to display the preprint in perpetuity. It is made available under aCC-BY 4.0 International license.

WRITE: intracellular concentrations only Not an ion channel; usually "Neither" (e.g., WRITE cai calcium accumulation mechanism)

Other terms that indicate neither type ARTIFICIAL CELL, Clamp, ELECTRODE, excessive installs/VERBATIM blocks, POINT_PROCESS gap, i=ic

File name / comments: Not reliable; "synaptic current" in comments ≠ receptor model could be a graded synapse; commented out names like "AMPAA" in a "GABAA" model

Fast K: Ambiguous; may mean K-A, K-DR, or K-UR → need context.

A-type: Default = A-type transient unless "slow"; may appear as Kv4, Afast, transient outward current, ITO.

K slow: Assign K-slow unless explicitly marked A-type slow or M-slow.

HH variant: Usually NaT or K-DR (sometimes K-A).

TTX sensitive: Assign NaT.

Anomalous rectifier / I-Funny / I-H: Assign I-H, not K-IR.

High threshold / High voltage: Assign HVA Ca current unless otherwise specific subtype specified (e.g., L-type).

Ligand-dependent: Likely a receptor (e.g, state transitions depend on neurotransmitter concentration)

Post-synaptic voltage-dependent: Likely an ion channel (e.g., depends on gating variables m/h/n)

Presynaptic voltage-dependent: Likely R Other (Rare) – graded synapse (e.g., conductance depends on vpre without NET RECEIVE)

Sodium, 1 m-gate: NaP.

Sodium, 1 h-gate: Ambiguous (flag).

Sodium, 2 gates: NaT (unless otherwise labeled, e.g., NaV1.9 → NaP).

Sodium, 3 gates: Na with slow inactivation.

Potassium, 1 n-gate: K-DR (Kv2/Kv3), unless explicitly labeled Kv1.1 (low threshold).

Potassium, 2 gates: K-A, unless explicitly Kv2.x → K-DR.

Potassium, 3+ gates: General K; refine if Kv4.x → A-type.

NaV1.9: NaP.

Kv1.x (e.g., 1.1, 1.2, 1.5, Shaker): I_KLT or D-type.

Kv2: K-DR.

Kv3: High-voltage K-DR.

Kv4 / Shal: A-type / ITO.

bioRxiv preprint doi: https://doi.org/10.64898/2026.04.23.720371; this version posted April 27, 2026. The copyright holder for this preprint (which was not certified by peer review) is the author/funder, who has granted bioRxiv a license to display the preprint in perpetuity. It is made available under aCC-BY 4.0 International license.

Kv7.x / KCNQ: M-type.

End by generating JSON of the form {"mechanisms": ["term1", "term2"], "type of model": "free text", "notes": "any notes of unusual things in the model"} where the terms (typically 0 or 1 but could be more represent the mechanism(s) (currents, pumps, and receptors) that are modeled in the file, chosen verbatim from the list below. When interpreting this list, HVA is high voltage activated, LT is low threshold, Rare should be interpreted as any other type of that channel that isn't covered by a more specific channel, "I Other (Rare)" is a current (e.g., Cl) that is not a Ca, K, or Na current, a prefix of R indicates a receptor (e.g., "R Other (Rare)" is a synaptic receptor that is not GABA, AMPA, or NMDA), and "Z Neither" is something that is neither a receptor nor a current (this could be a pump or it could be a utility file with many VERBATIM blocks that has no direct biological interpretation). Provide no additional explanation after the JSON."I Ca (HVA)", "I Ca (Rare)", "I Ca (T-type LT)", "I H", "I K (A-type)", "I K (Ca-activated)", "I K (Delayed Rectifier)", "I K (M-type)", "I K (Rare)", "I Na (Persistent)", "I Na (Rare)", "I Na (Slow inactivation)", "I Na (Transient)", "I Other (Rare)", "R GABA", "R Glutamate (AMPA)", "R Glutamate (NMDA)", "R Other (Rare)", "Z Neither"

Table S1. Annotation Heuristics for Manual Labeling of ModelDB Files

Heuristic Category	Indicator / Term	Annotation Mechanism Guidance
General Features	NET_RECEIVE	Likely receptor; discrete events rather than continuous current.
	POINT PROCESS	Likely receptor but could be some other localized mechanism (e.g. neither - graded synapse, neither - gap junction).
	SUFFIX	Likely ion channel but could be neither (e.g., SUFFIX NOTHING).
	WRITE intracellular concentrations only	Not an ion channel; usually “Neither” (e.g., WRITE cai calcium accumulation mechanism)
	Other terms that indicate neither type	ARTIFICIAL CELL, Clamp, ELECTRODE, excessive installs/VERBATIM blocks, POINT_PROCESS gap, i=ic
	File name / comments	Not reliable; “synaptic current” in comments ≠ receptor model could be a graded synapse; commented out names like “AMPAA” in a “GABAA” model
Comments / Mod-File Names	*Fast K*	Ambiguous; may mean K-A, K-DR, or K-UR → need context.
	A-type	Default = A-type transient unless “slow”; may appear as Kv4, Afast, transient outward current, ITO.
	K slow	Assign K-slow unless explicitly marked A-type slow or M-slow.

bioRxiv preprint doi: https://doi.org/10.64898/2026.04.23.720371; this version posted April 27, 2026. The copyright holder for this preprint (which was not certified by peer review) is the author/funder, who has granted bioRxiv a license to display the preprint in perpetuity. It is made available under aCC-BY 4.0 International license.

	HH variant	Usually NaT or K-DR (sometimes K-A).
	TTX sensitive	Assign NaT.
	Anomalous rectifier / I-Funny / I-H	Assign I-H, not K-IR.
	High threshold / High voltage	Assign HVA Ca current unless otherwise specific subtype specified (e.g., L-type).
Gating Dependence	*Ligand-dependent*	Likely a receptor (e.g, state transitions depend on neurotransmitter concentration)
	Post-synaptic voltage-dependent	Likely an ion channel (e.g., depends on gating variables m/h/n)
	Presynaptic voltage-dependent	Likely R Other (Rare) – graded synapse (e.g., conductance depends on vpre without NET RECEIVE)
Gating Variables	Sodium, 1 m-gate	NaP.
	Sodium, 1 h-gate	Ambiguous (flag).
	Sodium, 2 gates	NaT (unless otherwise labeled, e.g., NaV1.9 → NaP).
	Sodium, 3 gates	Na with slow inactivation.
	Potassium, 1 n-gate	K-DR (Kv2/Kv3), unless explicitly labeled Kv1.1 (low threshold).
	Potassium, 2 gates	K-A, unless explicitly Kv2.x → K-DR.
	Potassium, 3+ gates	General K; refine if Kv4.x → A-type.
Gene Mappings	NaV1.9	NaP.
	Kv1.x (e.g., 1.1, 1.2, 1.5, Shaker)	I_KLT or D-type.
	Kv2	K-DR.
	Kv3	High-voltage K-DR.
	Kv4 / Shal	A-type / ITO.
	Kv7.x / KCNQ	M-type.

bioRxiv preprint doi: https://doi.org/10.64898/2026.04.23.720371; this version posted April 27, 2026. The copyright holder for this preprint (which was not certified by peer review) is the author/funder, who has granted bioRxiv a license to display the preprint in perpetuity. It is made available under aCC-BY 4.0 International license.

Table S2. Feature List for XGBoost.

Feature	Drop	Type	Description
states_count		Text	State Count
clamp_yn		Text	Clamp Present
suffix_yn		Text	Suffix Present
point_process_yn		Text	Point Process
net_receive_yn		Text	Net Receive Mechanism
i_nonspecific_yn		Text	Nonspecific Current
not_ion_channel_yn		Text	Not an Ion Channel
not_receptor_yn		Text	Not a Receptor
has_mg_yn		Text	Has Magnesium
volt_min	Y	Text	Minimum Voltage
volt_max		Text	Maximum Voltage
has_include_yn		Text	Include block present
has_include_cap_yn		Text	Include block present in all capitals
read_ca_i_yn		Text	Reads intracellular Ca
read_ca_o_yn		Text	Reads extracellular Ca
read_cl_i_yn	Y	Text	Reads intracellular Cl
read_cl_o_yn	Y	Text	Reads extracellular Cl
read_e_ca_yn		Text	Reads E Ca
read_e_k_yn		Text	Reads E K
read_e_na_yn		Text	Reads E Na
read_e_other_yn		Text	Reads E Other
read_i_cal_yn		Text	Reads I Ca
read_i_cl_yn	Y	Text	Reads I Cl
read_i_k_yn	Y	Text	Reads I K
read_i_na_yn		Text	Reads I Na
read_i_other_yn	Y	Text	Reads I Other
read_k_i_yn		Text	Reads intracellular K

bioRxiv preprint doi: https://doi.org/10.64898/2026.04.23.720371; this version posted April 27, 2026. The copyright holder for this preprint (which was not certified by peer review) is the author/funder, who has granted bioRxiv a license to display the preprint in perpetuity. It is made available under aCC-BY 4.0 International license.

read_k_o_yn		Text	Reads extracellular K
read_na_i_yn		Text	Reads intracellular Na
read_na_o_yn	Y	Text	Reads extracellular Na
read_other_i_yn	Y	Text	Reads intracellular Other
read_other_o_yn	Y	Text	Reads extracellular Other
write_ca_i_yn		Text	Writes intracellular Ca
write_ca_o_yn	Y	Text	Writes extracellular Ca
write_cl_i_yn	Y	Text	Writes intracellular Cl
write_cl_o_yn	Y	Text	Writes extracellular Cl
write_i_cal_yn		Text	Writes I Ca
write_i_cl_yn	Y	Text	Writes I Cl
write_i_k_yn		Text	Writes I K
write_i_na_yn		Text	Writes I Na
write_i_other_yn		Text	Writes I Other
write_k_i_yn	Y	Text	Writes intracellular K
write_k_o_yn	Y	Text	Writes extracellular K
write_na_i_yn	Y	Text	Writes intracellular Na
write_na_o_yn	Y	Text	Writes extracellular Na
write_other_i_yn	Y	Text	Writes intracellular Other
write_other_o_yn	Y	Text	Writes extracellular Other
write_unknown_yn	Y	Text	Writes I Unknown
voltage_simfeat	Y	Text	Voltage Trace
v_interval1_time_to_90_max_simfeat		Sim	Voltage Interval 1 Time to 90% of Maximum
v_interval1_time_to_90_min_simfeat	Y	Sim	Voltage Interval 1 Time to 90% of Minimum
v_interval1_time_min_to_90_max_simfeat	Y	Sim	Voltage Interval 1 Time from Minimum to 90% of Maximum
v_interval1_time_max_to_90_min_simfeat	Y	Sim	Voltage Interval 1 Time from Maximum to 90% of Minimum
v_interval1_initial_val_simfeat		Sim	Voltage Interval 1 Initial Value

bioRxiv preprint doi: https://doi.org/10.64898/2026.04.23.720371; this version posted April 27, 2026. The copyright holder for this preprint (which was not certified by peer review) is the author/funder, who has granted bioRxiv a license to display the preprint in perpetuity. It is made available under aCC-BY 4.0 International license.

v_interval1_max_val_simfeat		Sim	Voltage Interval 1 Maximum Value
v_interval1_min_val_simfeat	Y	Sim	Voltage Interval 1 Minimum Value
v_interval2_time_to_90_recovery_simfeat		Sim	Voltage Interval 2 Time to 90% Recovery
v_interval2_recovery_start_val_simfeat		Sim	Voltage Interval 2 Recovery Start Value
v_interval2_final_val_simfeat		Sim	Voltage Interval 2 Final Value
v_interval2_max_val_simfeat	Y	Sim	Voltage Interval 2 Maximum Value
v_interval2_min_val_simfeat		Sim	Voltage Interval 2 Minimum Value
ik_interval1_time_to_90_max_simfeat		Sim	I K Interval 1 Time to 90% of Maximum
ik_interval1_time_to_90_min_simfeat		Sim	I K Interval 1 Time to 90% of Minimum
ik_interval1_time_min_to_90_max_simfeat	Y	Sim	I K Interval 1 Time from Minimum to 90% of Maximum
ik_interval1_time_max_to_90_min_simfeat		Sim	I K Interval 1 Time from Maximum to 90% of Minimum
ik_interval1_initial_val_simfeat	Y	Sim	I K Interval 1 Initial Value
ik_interval1_max_val_simfeat		Sim	I K Interval 1 Maximum Value
ik_interval1_min_val_simfeat		Sim	I K Interval 1 Minimum Value
ik_interval2_time_to_90_recovery_simfeat		Sim	I K Interval 2 Time to 90% Recovery
ik_interval2_recovery_start_val_simfeat	Y	Sim	I K Interval 2 Recovery Start Value
ik_interval2_final_val_simfeat	Y	Sim	I K Interval 2 Final Value
ik_interval2_max_val_simfeat		Sim	I K Interval 2 Maximum Value
ik_interval2_min_val_simfeat		Sim	I K Interval 2 Minimum Value
ina_interval1_time_to_90_max_simfeat		Sim	I Na Interval 1 Time to 90% of Maximum
ina_interval1_time_to_90_min_simfeat		Sim	I Na Interval 1 Time to 90% of Minimum
ina_interval1_time_min_to_90_max_simfeat		Sim	I Na Interval 1 Time from Minimum to 90% of Maximum
ina_interval1_time_max_to_90_min_simfeat		Sim	I Na Interval 1 Time from Maximum to 90% of Minimum
ina_interval1_initial_val_simfeat		Sim	I Na Interval 1 Initial Value
ina_interval1_max_val_simfeat	Y	Sim	I Na Interval 1 Maximum Value

bioRxiv preprint doi: https://doi.org/10.64898/2026.04.23.720371; this version posted April 27, 2026. The copyright holder for this preprint (which was not certified by peer review) is the author/funder, who has granted bioRxiv a license to display the preprint in perpetuity. It is made available under aCC-BY 4.0 International license.

ina_interval1_min_val_simfeat		Sim	I Na Interval 1 Minimum Value
ina_interval2_time_to_90_recovery_simfeat		Sim	I Na Interval 2 Time to 90% Recovery
ina_interval2_recovery_start_val_simfeat	Y	Sim	I Na Interval 2 Recovery Start Value
ina_interval2_final_val_simfeat		Sim	I Na Interval 2 Final Value
ina_interval2_max_val_simfeat	Y	Sim	I Na Interval 2 Maximum Value
ina_interval2_min_val_simfeat		Sim	I Na Interval 2 Minimum Value
ica_interval1_time_to_90_max_simfeat		Sim	I Ca Interval 1 Time to 90% of Maximum
ica_interval1_time_to_90_min_simfeat		Sim	I Ca Interval 1 Time to 90% of Minimum
ica_interval1_time_min_to_90_max_simfeat		Sim	I Ca Interval 1 Time from Minimum to 90% of Maximum
ica_interval1_time_max_to_90_min_simfeat		Sim	I Ca Interval 1 Time from Maximum to 90% of Minimum
ica_interval1_initial_val_simfeat		Sim	I Ca Interval 1 Initial Value
ica_interval1_max_val_simfeat		Sim	I Ca Interval 1 Maximum Value
ica_interval1_min_val_simfeat		Sim	I Ca Interval 1 Minimum Value
ica_interval2_time_to_90_recovery_simfeat		Sim	I Ca Interval 2 Time to 90% Recovery
ica_interval2_recovery_start_val_simfeat	Y	Sim	I Ca Interval 2 Recovery Start Value
ica_interval2_final_val_simfeat		Sim	I Ca Interval 2 Final Value
ica_interval2_max_val_simfeat		Sim	I Ca Interval 2 Maximum Value
ica_interval2_min_val_simfeat		Sim	I Ca Interval 2 Minimum Value

bioRxiv preprint doi: https://doi.org/10.64898/2026.04.23.720371; this version posted April 27, 2026. The copyright holder for this preprint (which was not certified by peer review) is the author/funder, who has granted bioRxiv a license to display the preprint in perpetuity. It is made available under aCC-BY 4.0 International license.

Table S3. Pairwise agreement.

Counts and percent agreement between model predictions across the full dataset.

Model 1	Model 2	N	Agree n (%)	Disagree n (%)	Kappa
GPT-5.2 (run 1)	GPT-5.2 (run 2)	5133	4899 (95.4%)	234 (4.6%)	0.95
GPT-5.2 (run 1)	GPT-5.2 + heuristics	5133	4713 (91.8%)	420 (8.2%)	0.91
GPT-5.2 (run 2)	GPT-5.2 + heuristics	5133	4695 (91.5%)	438 (8.5%)	0.91
GPT-5.2 + heuristics	GPT-mini + heuristics	5133	4618 (90.0%)	515 (10.0%)	0.89
GPT-mini	GPT-mini + heuristics	5132	4617 (90.0%)	515 (10.0%)	0.89
GPT-5.2 (run 2)	GPT-mini + heuristics	5133	4546 (88.6%)	587 (11.4%)	0.87
GPT-5.2 (run 1)	GPT-mini + heuristics	5133	4542 (88.5%)	591 (11.5%)	0.87
GPT-5.2 (run 2)	GPT-mini	5132	4539 (88.4%)	593 (11.6%)	0.87
GPT-5.2 (run 1)	GPT-mini	5132	4527 (88.2%)	605 (11.8%)	0.87
GPT-5.2 + heuristics	GPT-mini	5132	4432 (86.4%)	700 (13.6%)	0.85

Table S4. Run-to-run agreement vs. human annotation confidence.

Agreement between repeated runs of each model for high- and lower-confidence cases.

Model (run-to-run)	High Confidence (n=922)	Low Confidence (n=177)	Overall (n=1099)
GPT-5.2 (run 1 vs. run 2)	879 (95.3%)	167 (94.4%)	1046 (95.2%)
GPT-5.2 vs. GPT-5.2 + heuristics	849 (92.1%)	160 (90.4%)	1009 (91.8%)
GPT-mini vs. GPT-mini + heuristics	839 (91.0%)	145 (81.9%)	984 (89.5%)
GPT-5.2 vs. GPT-mini	833 (90.3%)	141 (79.7%)	974 (88.6%)

bioRxiv preprint doi: https://doi.org/10.64898/2026.04.23.720371; this version posted April 27, 2026. The copyright holder for this preprint (which was not certified by peer review) is the author/funder, who has granted bioRxiv a license to display the preprint in perpetuity. It is made available under aCC-BY 4.0 International license.

Figure S1. Confusion Matrices.

Type and subtype confusion matrices for XGBoost (left), GPT without heuristics (middle), and GPT with heuristics.

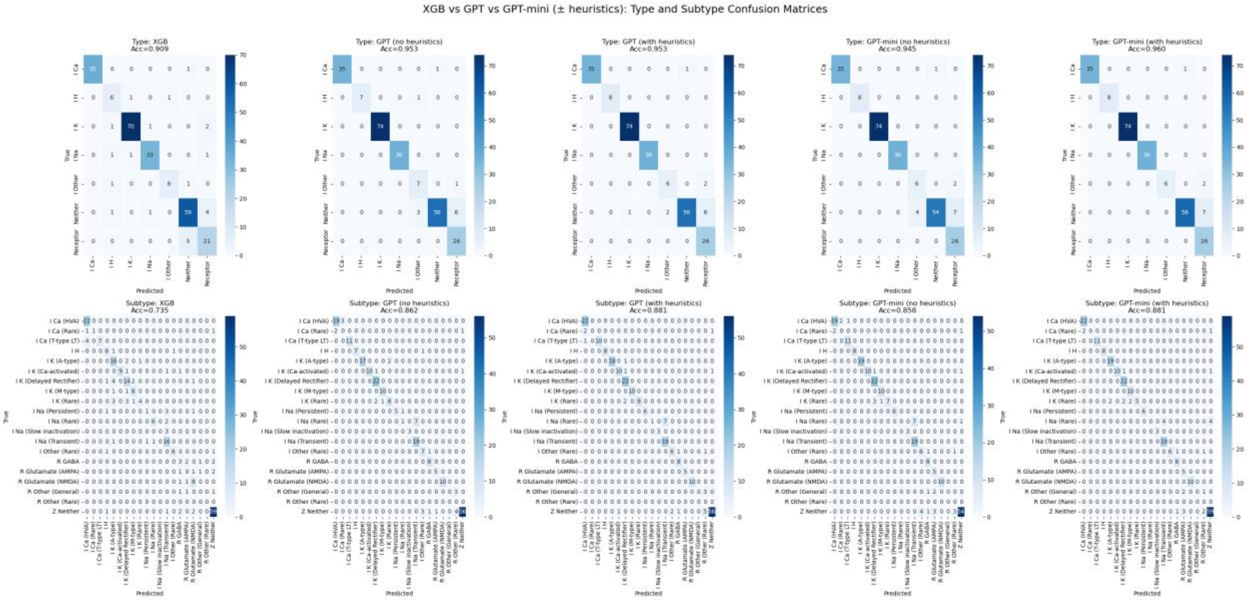

Figure S2. Correct Classification Overlap.

UpSet plot showing overlap in correctly classified files across XGBoost and GPT models. Filled dots indicate models included in each intersection, and horizontal bars show the total number of correctly classified files per model. Vertical bars represent the number of files in each intersection.

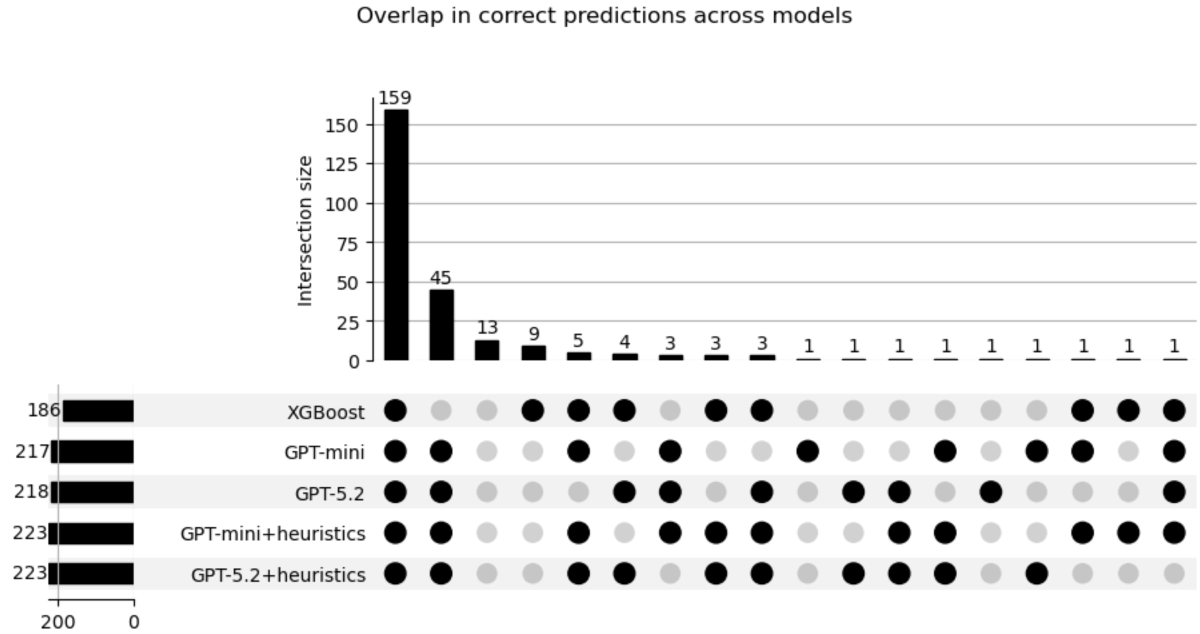

bioRxiv preprint doi: https://doi.org/10.64898/2026.04.23.720371; this version posted April 27, 2026. The copyright holder for this preprint (which was not certified by peer review) is the author/funder, who has granted bioRxiv a license to display the preprint in perpetuity. It is made available under aCC-BY 4.0 International license.

Figure S3. XGBoost Feature Importance.

(A) Top 15 features by ranked by XGBoost importance. (B) SHAP summary plot showing the impact of individual features on model output.

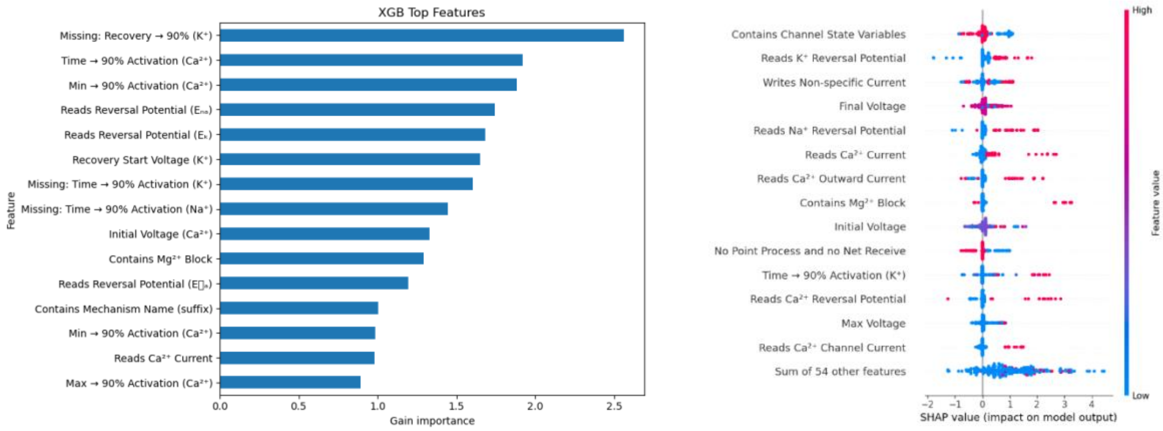

Supplement: Supplement 1 [file NIHPP2026.04.23.720371v1-supplement-1.pdf]
